# The Usefulness of Endothelial Cell Adhesion Molecules and Anti-C1q Antibody in Monitoring Systemic Lupus Erythematosus Disease Activity

**DOI:** 10.1155/2014/275194

**Published:** 2014-10-29

**Authors:** Hasni Mahayidin, Nurul Khaiza Yahya, Wan Syamimee Wan Ghazali, Asmahan Mohd Ismail, Wan Zuraida Wan Ab Hamid

**Affiliations:** ^1^Department of Pathology, Faculty of Medicine and Health Sciences, Universiti Putra Malaysia (UPM), 43400 Serdang, Selangor, Malaysia; ^2^Department of Immunology, School of Medical Sciences, Universiti Sains Malaysia, Health Campus, 16150 Kubang Kerian, Kelantan, Malaysia; ^3^Department of Medicine, Hospital Universiti Sains Malaysia, 16150 Kubang Kerian, Kelantan, Malaysia; ^4^Department of Medicine, Hospital Raja Perempuan Zainab II, 15000 Kota Bharu, Kelantan, Malaysia

## Abstract

*Objectives.* The study was conducted to determine the correlation of ICAM-1, VCAM-1, and anti-C1q antibody levels with SLE disease activity index (SLEDAI) and standard SLE disease activity immunological markers (anti-dsDNA and sera C3 and C4).* Study Design.* This was a cross-sectional study.* Materials and Methods.* Blood samples were obtained from 95 SLE patients (45 active SLE and 50 nonactive SLE) and 50 controls. The subjects were assessed using SLEDAI and score of more than five is determined as having active SLE. The sera were tested for serum ICAM-1, VCAM-1, and anti-C1q (ELISA), anti-dsDNA (CLIFT), serum C3, and serum C4 (immunonephelometry).* Results.* Anti-dsDNA and anti-C1q antibody showed good positive correlations with SLEDAI (*r* = 0.529, *P* < 0.001 and *r* = 0.559, *P* < 0.001, resp.). VCAM-1 and sera C3 and C4 showed fair correlation with SLEDAI (*r* = 0.294, *P* = 0.004; *r* = −0.312, *P* = 0.002; and *r* = −0.382, *P* < 0.001, resp.). ICAM-1 level showed no significant correlation with SLEDAI (*P* = 0.062). There were significant correlations of VCAM-1 and anti-C1q antibody with anti-dsDNA (*r* = 0.226, *P* = 0.006 and *r* = 0.511, *P* < 0.001, resp.). VCAM-1 showed poor inverse correlation with serum C3 (*r* = −0.183, *P* = 0.028) and fair inverse correlation with serum C4 (*r* = −0.251, *P* = 0.002). Anti-C1q antibody demonstrated fair inverse correlation with both sera C3 and C4 (*r* = −0.420, *P* ≤ 0.001 and *r* = −0.398, *P* < 0.001, resp.). However, ICAM-1 showed no significant correlation with anti-dsDNA and sera C3 and C4 (*P* = 0.259, *P* = 0.626 and *P* = 0.338, resp.).* Conclusions.* The serum levels of anti-C1q antibody in SLE patients showed the best correlation with the SLEDAI and standard immunological tests for SLE disease activity. These data support that anti-C1q antibody is a useful marker for monitoring SLE global disease activity. The potential of VCAM-1 needs further confirmation.

## 1. Introduction

SLE is a systemic autoimmune disorder characterized by presence of autoantibodies against self-nuclear components that resulted in chronic inflammation of various tissues and organs [1]. The disease activity monitoring is important for SLE management and it may be difficult to distinguish flares from permanent changes that have occurred due to chronic irreversible damage. Scoring systems which comprise a variety of clinical and laboratory parameters have been created to assess SLE disease activity including BILAG, SLEDAI, SLAM, LAI, and ECLAM [2, 3].

Currently, anti-dsDNA is widely used in assessing SLE disease activity apart from serum C3, serum C4, and C-reactive protein (CRP) levels [4]. Anti-dsDNA, serum C3, and serum C4 levels are among the laboratory parameters which are included in the SLEDAI. However, there is substantial percentage of patients who are persistently negative for anti-dsDNA, thus halting proper monitoring of the disease activity. Depending on studies, the percentage of SLE with negative anti-dsDNA can be as high as 20% [5]. Apart from that, studies on associations of various autoantibodies particularly anti-dsDNA and complement proteins with disease activity in SLE have shown inconsistent results. Such findings have led to revisiting of the value of the conventional biomarkers which are widely used in disease activity indices [6].

ICAM-1 and VCAM-1 are immunoglobulin superfamily of endothelial cell adhesion molecules which are important for leukocyte transmigration from within blood vessel into extravascular site of inflammatory process. ICAM-1 is lowly expressed on the surface of endothelial cells and antigen presenting cells. However, the expression is greatly increased by cytokines stimulation, that is, IL-1, IFN-*γ*, and TNF-*α*, especially during inflammatory responses [7, 8]. VCAM-1 is not expressed by naïve endothelial cell culture, but exposure to inflammatory cytokines IL-1, TNF-*α*, and IFN-*γ* results in its rapid upregulation. It is involved in extravasation of chemokine-recruited lymphocytes and monocytes from circulation into the inflammatory tissue sites by binding to integrin *α*4*β*1, or very late activation molecule 4 (VLA-4) whose expression is increased during conversion of antigen-naïve T cells to effector T cells [8]. VCAM-1 levels sustained high level in two to three hours and gradually diminished over several days [9]. ICAM-1 has been studied in various conditions including autoimmune and inflammatory conditions including SLE. However, it has shown inconsistent results where there were studies that found that ICAM-1 is useful for SLE disease activity assessment and prediction [10, 11], while other studies have shown contrary results [12, 13]. VCAM-1 levels have been found to be elevated in SLE and associated with the disease activity. Some studies have shown that it is statistically significant in some organ-specific SLE such as lupus nephritis, vasculitis, and haematological involvements [11, 12, 14].

C1q is the first component of the classical pathway that is activated by the immune complexes. Presence of anti-C1q antibody results in delay of immune complexes clearance in some conditions including SLE [15]. The anti-C1q antibody is strongly associated with lupus nephritis, which is one of the most serious complications of SLE [16–18]. However, some studies have shown that anti-C1q antibody is associated with SLE global activity but not specifically with active lupus nephritis [19, 20].

The aim of this study is to determine the correlation of ICAM-1, VCAM-1, and anti-C1q antibody levels with SLE disease activity index (SLEDAI) and standard SLE disease activity immunological markers (anti-dsDNA and sera C3 and C4). The data obtained from this study is useful to verify the reliability of ICAM-1 and VCAM-1 as SLE disease monitoring and flare predictor tools and the potential of anti-C1q antibody as SLE global activity biomarker [19].

## 2. Methodology 

This was a cross-sectional study involving SLE patients in two medical centres in Kelantan, that is, Hospital Universiti Sains (USM), Kubang Kerian, and Hospital Raja Perempuan Zainab II (HRPZ II), Kota Bharu, between the period of June 2012 and September 2013. 95 (45 active and 50 nonactive) SLE patients who fulfilled the ACR classification criteria for SLE were included in this study. The nonactive SLE cases from outpatient clinics were selected by simple random sampling method. Due to the difficulty in getting the active SLE cases, universal sampling method was applied where every consented patient that fulfilled the criteria was included. In addition, 50 apparently healthy individuals were included as controls. All participants were informed about the study and gave written consent.

The disease activities were evaluated according to the SLEDAI (SELENA modification) and patients with score above five are defined as having active disease [21, 22]. Patients who aged less than 13 years old and were HIV positive, pregnant, and having acute infections and chronic illnesses, for example, diabetes mellitus, malignancies, and tuberculosis, were excluded.

The patients' demographic and relevant clinical data including status of lupus nephritis were obtained from the medical records. The peripheral blood samples were obtained and allowed to clot prior to centrifugation. The sera were aliquot and stored at −80°C until respective analysis was carried out. Serum ICAM-1 and VCAM-1 levels were measured by using precoated ELISA kit (Cusabio, China) that employed the sandwich enzyme immunoassay technique for quantitative measurement of human ICAM-1 and VCAM-1 concentrations. The anti-C1q antibody levels were measured using precoated ELISA kit (Orgentec, Germany) that quantitatively measures the IgG subclass of antibodies against C1q in human serum or plasma. Analyses were carried out according to manufacturers' protocols. Anti-dsDNA was measured semiquantitatively using Fluoro nDNA test (MBL, Japan) that employed* Crithidia luciliae* indirect immunofluorescent test (CLIFT) method. Anti-dsDNA with titer 1 : 10 was considered positive and the results were reported in titer from 1 : 10 to 1 : 160. Sera C3 and C4 levels analysis was done using quantitative determination by immunonephelometry (BN-ProSpec, Siemens, USA). Sera C3 and C4 levels were taken as low at the level of less than 0.66 g/L and 0.20 g/L, respectively.

## 3. Statistical Analysis

Data analysis were done using SPSS version 20. The mean with standard deviation (SD) and median with interquartile range (IQR) were determined depending on data distribution. Shapiro-Wilk's test was used to evaluate the distribution. The mean or median differences of serum markers in active SLE patients, nonactive SLE patients, and healthy controls were performed using one-way ANOVA and Kruskal-Wallis test, respectively. Post hoc test Scheffe's procedure or Mann-Whitney test with Bonferroni correction was used to compare the variables values. The correlations tests were carried out using Pearson's correlation coefficient or Spearman's rank correlation coefficient. Comparisons and correlations were considered significant when *P* < 0.05.

## 4. Result 

### 4.1. Demographic Data

95 subjects were enrolled in this study, which consists of 45 active SLE patients and 50 nonactive SLE patients. The demographic data of the subjects is shown in Table 1. Out of 95 SLE patients, 55 (57.9%) patients had lupus nephritis while the other 40 (42.1%) patients did not.

### 4.2. Prevalence of Standard Disease Activity Immunological Markers

Anti-dsDNA was positive in 29 (64.4%) of active SLE patients and 12 (24.0%) of nonactive SLE patients. Twenty-six (57.8%) of active SLE patients had low serum C3 levels. Only 6 (12%) of nonactive SLE patients and 1 (2%) of controls had low serum C3 levels. Low serum C4 levels in active SLE and nonactive SLE patients were 33 (73.3%) and 24 (48%), respectively. Thirteen (26%) of controls had low serum C4 levels.

### 4.3. Comparison between Levels of Serum Markers in Active SLE, Nonactive SLE, and Control Groups

There were statistically significant difference of VCAM-1 and anti-C1q antibody levels among active SLE, nonactive SLE, and control groups with *P* < 0.001. However, there was no significant difference of ICAM-1 level between the three groups (*P* = 0.107) (Table 2).

### 4.4. Correlation between Levels of Serum Markers and SLEDAI Score in SLE Patients

Anti-dsDNA and anti-C1q antibody showed good positive correlation with SLEDAI with Spearman's rho of 0.529 (*P* < 0.001) and 0.559 (*P* < 0.001), respectively. VCAM-1 only showed fair positive correlation with SLEDAI score. Both sera C3 and C4 demonstrated fair inverse correlation while ICAM-1 level showed no significant correlation with SLEDAI score (Table 3).

### 4.5. Correlation between Levels of ICAM-1, VCAM-1, and Anti-C1q Antibody with Standard SLE Disease Activity Immunological Markers

ICAM-1 showed no significant correlation with anti-dsDNA level, serum C3 level, and serum C4 level. Both VCAM-1 and anti-C1q antibody showed significant correlation with anti-dsDNA, serum C3, and serum C4 levels (Table 4).

## 5. Discussion 

Anti-dsDNA, serum C3, and serum C4 levels are widely acceptable standard immunological markers in evaluating disease activity in SLE. Anti-dsDNA using CLIFT method, due to its semiquantitative test, is less sensitive for disease monitoring compared to ELISA method. However, many clinical laboratories still opt for CLIFT method due to its high specificity for confirmation of SLE diagnosis. Positive anti-dsDNA in SLE patients ranged from 36 to 69% [23]. Negative anti-dsDNA levels could be due to nonactive (stable) state of SLE although it was documented that around 20% of SLE patients were persistently negative for anti-dsDNA [5]. In this study, the anti-dsDNA levels were positive in 41 (43.16%) of SLE patients and the percentage was higher in active SLE than in nonactive SLE. Anti-dsDNA gave no false positive result in this study and this finding agreed with previous studies using CLIFT method. The use of ELISA for anti-dsDNA might give better sensitivity but with reduced specificity in SLE [24].

More than half of active SLE patients had low sera C3 and C4 levels with higher percentage seen in serum C4 (73.3%) than in serum C3 (57.8%). However, almost half of nonactive SLE patients also had low serum C4 levels and this finding showed that serum C4 levels were not that useful in determining SLE disease activity status. Serum C3 was better in reflecting SLE disease activity status as only 12.0% of nonactive SLE patients and 2.0% of controls had low serum C3.

In this study, the correlation of anti-dsDNA with SLEDAI score was better than serum C3, serum C4, ICAM-1, and VCAM-1. However, its correlation was weaker than correlation of anti-C1q, even though both anti-dsDNA and anti-C1q demonstrated good positive correlation with SLEDAI score. These similar findings were also shown in other studies [11, 20, 25].

Both serum C3 and serum C4 levels showed fair inverse correlation with SLE disease activity. This reflected complement activation in SLE pathogenesis and at the same time supported the use of anti-C1q antibody as a useful marker for SLE disease monitoring. Complement system is important for clearing up the antigen-antibody complexes, whose formation was increased in SLE flares. The presence of autoantibody against C1q would impair the classical pathway of the complement cascade which is crucial for immune complexes clearance. If comparison between sera C3 and C4 levels was to be evaluated, serum C4 levels showed better correlation with SLEDAI score. However, according to this study, the serum C3 and serum C4 correlation with SLEDAI score were weaker than anti-C1q antibody.

VCAM-1 showed fair correlation with SLEDAI score. Even though the correlation was significant, it was weaker than anti-C1q antibody, anti-dsDNA, serum C3, and serum C4. ICAM-1 level, on the other hand, did not correlate significantly with SLE disease activity. Many studies agreed that ICAM-1 did not reflect the disease activity in SLE patients [12, 13, 26].

In this study, the ICAM-1 levels did not correlate significantly with anti-dsDNA, serum C3, and serum C4 levels. These findings were expected as ICAM-1 level also showed no significant correlation with SLEDAI score. A study in Cairo with similar method had found that there was no correlation between ICAM-1 and anti-dsDNA and serum C3 in SLE patients [11]. That particular study demonstrated similar findings for VCAM-1 as well. That was in contrast to our recent findings where VCAM-1 showed significant positive correlation with anti-dsDNA and significant negative correlation with serum C3 and serum C4. Better correlation was seen between VCAM-1 and serum C4 compared to serum C3 levels. This was expected in view that serum C4 levels demonstrated better correlation with SLEDAI score than serum C3 in this study.

One of the earlier studies on adhesion molecules showed similar findings to this current study; that is, VCAM-1 levels were correlated with SLE disease activity and anti-dsDNA and inversely correlated with serum C3. These findings were consistent with the theory that immune complex formation and upregulation of adhesion molecules are involved in SLE pathogenesis [12]. An in vitro study has demonstrated that anti-dsDNA was able to enhance the expression of endothelial cell adhesion molecules in SLE [27].

In this study, anti-C1q antibody levels were significantly different between the three groups of active SLE, nonactive SLE, and controls (Table 2). According to the cut-off value given by the manufacturer (10 U/mL), it was found that 64.4% of active SLE patients had high anti-C1q antibody levels compared to only 18% of nonactive SLE and 10.0% of controls. These findings were similar to other studies that had found higher prevalence of anti-C1 antibody in SLE than in healthy controls [19, 28, 29]. The prevalence of anti-C1q antibody in this study was 40.0% and this was in accordance with studies done in Brazil (39.5%) and India (58.3%). The discrepancies found might be due to differences in population studied, as well as in variations of the ELISA kits used [22]. Anti-C1q antibody was also found in healthy population and the prevalence ranges between 2.0 and 8.0% [30]. In this study, the prevalence of anti-C1q antibody in controls was slightly higher (10.0%).

Anti-C1q antibody showed good correlation with anti-dsDNA and it was better correlated than VCAM-1. Anti-C1q antibody also showed significant moderate correlation with serum C3 and serum C4 levels. Interestingly, better correlation was seen between anti-C1q antibody and serum C3 compared to serum C4, as demonstrated by VCAM-1. This discrepancy between findings of sera C3 and C4 cannot be clearly explained. Other studies had also yielded conflicting results regarding relation of SLE disease activity with levels of sera C3 and C4. Some studies have found that serum C3 was superior in SLE disease monitoring, while some demonstrated the same for serum C4 [31–33].

Many of previous studies have found that anti-C1q antibody was a useful marker for lupus nephritis [16–18]. However, there were also studies that proved anti-C1q antibody did not significantly associate with lupus nephritis [19, 20, 29]. This current study showed that there was no significant difference between anti-C1q antibody levels in lupus nephritis and non-lupus nephritis patients (*Z* = −1.25, *P* = 0.211).

## 6. Summary

The serum levels anti-C1q antibody showed the best correlation with SLEDAI score, comparable to anti-dsDNA. Anti-C1q antibody also showed significant correlation with standard immunological tests for SLE disease activity (anti-dsDNA and sera C3 and C4). These results supported the fact that anti-C1q antibody may serve as a useful marker for monitoring SLE global disease activity. The potential of serum VCAM-1 needs to be confirmed with longitudinal studies in the future.

## 7. Recommendation

Majority of the SLE patients were on immunosuppressant and each patient was on different type of medications and dosage. It was impossible to standardize this issue and it cannot be ruled out that these medications might have different effects on the markers of interest. Future study which can address this issue would very much contribute to elucidate this matter.

## Figures and Tables

**Table 1:** Demographic data of active SLE, nonactive SLE, and controls.

Characteristics	Active SLE *n* = 45 *n* (%)	Nonactive SLE *n* = 50 *n* (%)	Controls *n* = 50 *n* (%)	*P* value
Age (years)	26.00 (8.57)^a^	31.32 (8.63)^a^	31.44 (8.86)^a^	**0.003** ^ b^
<20	12 (26.7)	6 (12.0)	0 (0.0)	
20–35	26 (57.8)	27 (54.0)	37 (74.0)	
>35	7 (15.5)	17 (34.0)	13 (26.0)	
Sex				
Female	42 (93.3)	49 (98)	42 (84.0)	—
Male	3 (6.7)	1 (2)	8 (16.0)	
Race				
Malay	44 (97.8)	46 (92.0)	42 (84.0)	—
Chinese	0 (0.0)	3 (6.0)	5 (10.0)	
Indians	0 (0.0)	0 (0.0)	3 (6.0)	
Others	1 (2.2)	1 (2.0)	0 (0.0)	
Disease duration (years)	1.00 (2.75)^c^	6.50 (7.50)^c^	—	**<0.001** ^ d^
SLEDAI score	12.00 (14.00)^c^	2.00 (2.00)^c^	—	**<0.001** ^ e^

^a^Mean (SD).

^b^One-way ANOVA. Post hoc test Scheffe's procedure: active SLE versus nonactive SLE (*P* = 0.014), active SLE versus control (*P* = 0.011), and nonactive SLE versus control (*P* = 0.998).

^c^Median (IQR).

^d^Mann-Whitney test, *Z* statistic = −5.69.

^e^Mann-Whitney test, *Z* statistic = −8.46.

Level of significance is set at *P* < 0.05.

**Table 2:** Summary of comparison between levels of serum markers in active SLE, nonactive SLE, and control groups.

Variables	Active SLE (*n* = 45) Median (IQR)	Nonactive SLE (*n* = 50) Median (IQR)	Control (*n* = 50) Median (IQR)	*X* ^2^ statistic (df)	*P* value^a^
ICAM-1 (ng/mL)	194.41 (330.48)	146.62 (88.11)	109.68 (190.30)	4.46 (2)	0.107

VCAM-1 (ng/mL)	34.53^a^ (25.83)	27.75^a^ (20.76)	14.09^a^ (25.12)	36.88 (2)	**<0.001**

Anti-C1q (U/mL)	19.67^a^ (43.59)	2.75^a^ (5.69)	1.47^a^ (2.64)	42.47 (2)	**<0.001**

^a^Statistically significant difference between pairs at *P* < 0.05 by post hoc test Bonferroni's procedure.

**Table 3:** Correlation between levels of serum markers and SLEDAI score in SLE patients.

Variables	SLEDAI (score), (*n* = 95)	
	*r*	*P* value
Anti-dsDNA (titer)	0.529^a^	**<0.001**
Serum C3 (g/L)	−0.312^b^	**0.002**
Serum C4 (g/L)	−0.382^a^	**<0.001**
ICAM-1 (ng/mL)	0.192^a^	0.062
VCAM-1 (ng/mL)	0.294^a^	**0.004**
Anti-C1q (U/mL)	0.559^a^	**<0.001**

^a^Spearman's rank correlation coefficient, level of significance is *P* < 0.05.

^b^Pearson's correlation coefficient, level of significance is *P* < 0.05.

**Table 4:** Correlations of ICAM-1, VCAM-1, and anti-C1q antibody with standard SLE disease activity immunological markers.

Variables	Anti-dsDNA (*n* = 145)		Serum C3 (*n* = 145)			Serum C4 (*n* = 145)	
	*r* ^a^	*P* value		*r* ^b^	*P* value	*r* ^a^	*P* value
ICAM-1	0.094	0.259		−0.041	0.626	0.080	0.338
VCAM-1	0.226	**0.006**		−0.183	**0.028**	−0.251	**0.002**
Anti-C1q	0.511	**<0.001**		−0.420	**<0.001**	−0.398	**<0.001**

^a^Spearman's rank correlation coefficient, level of significance is *P* < 0.05.

^b^Pearson's correlation coefficient, level of significance is *P* < 0.05.
